# A multicomponent exercise intervention to improve physical functioning, cognition and psychosocial well-being in elderly nursing home residents: a study protocol of a randomized controlled trial in the PROCARE (prevention and occupational health in long-term care) project

**DOI:** 10.1186/s12877-019-1386-6

**Published:** 2019-12-23

**Authors:** Thomas Cordes, Laura L. Bischoff, Daniel Schoene, Nadja Schott, Claudia Voelcker-Rehage, Charlotte Meixner, Luisa-Marie Appelles, Michael Bebenek, Andre Berwinkel, Claudia Hildebrand, Thomas Jöllenbeck, Bettina Johnen, Wolfgang Kemmler, Thomas Klotzbier, Heide Korbus, Julian Rudisch, Lutz Vogt, Matthias Weigelt, Rita Wittelsberger, Katharina Zwingmann, Bettina Wollesen

**Affiliations:** 1 Department of Human Movement Science, University of Hamburg, Hamburg, Germany; 2 Institute of Medical Physics, Friedrich-Alexander University of Erlangen-Nürnberg, Erlangen, Germany; 3 Department of Sports and Exercise Science, University of Stuttgart, Stuttgart, Germany; 4 Institute of Human Movement Science and Health, Chemnitz University of Technology, Chemnitz, Germany; 5 Institute of Sports and Sports Science, Karlsruhe Institute of Technology, Karlsruhe, Germany; 6 Department of Sport & Health Sciences, University of Paderborn, Paderborn, Germany; 7 Institute of Sports Sciences, Goethe-University Frankfurt, Frankfurt, Germany

**Keywords:** Nursing home, Intervention, Physical activity, Exercise, Cognition, Frailty, Aged, ADL, Physical function, Clinical trial

## Abstract

**Background:**

Older adults, who are living in nursing homes that provide a high level of long-term nursing care, are characterized by multimorbidity and a high prevalence of dependency in activities of daily living. Results of recent studies indicate positive effects of structured exercise programs during long-term care for physical functioning, cognition, and psychosocial well-being. However, for frail elderly the evidence remains inconsistent. There are no evidence-based guidelines for exercises for nursing home residents that consider their individual deficits and capacities. Therefore, high-quality studies are required to examine the efficacy of exercise interventions for this multimorbid target group. The purpose of this study is to determine the feasibility and efficacy of a multicomponent exercise intervention for nursing home residents that aims to improve physical and cognitive functioning as well as quality of life.

**Methods:**

A two-arm single-blinded multicenter randomized controlled trial will be conducted, including 48 nursing homes in eight regions of Germany with an estimated sample size of 1120 individuals. Participants will be randomly assigned to either a training or a waiting time control group. For a period of 16 weeks the training group will meet twice a week for group-based sessions (45–60 min each), which will contain exercises to improve physical functioning (strength, endurance, balance, flexibility) and cognitive-motor skills (dual-task). The intervention is organized as a progressive challenge which is successively adapted to the residents’ capacities. Physical functioning, cognitive performance, and quality of life will be assessed in both study groups at baseline (pre-test), after 16-weeks (post-treatment), and after 32-weeks (retention test, intervention group only).

**Discussion:**

This study will provide information about the efficacy of a multicomponent exercise program in nursing homes (performance, recruitment). Results from this trial will contribute to the evidence of multicomponent exercises, which specifically focus on cognitive-motor approaches in the maintenance of mental and physical functioning. In addition, it will help to encourage older adults to actively engage in social life. Furthermore, the findings will lead to recommendations for health promotion interventions for frail nursing home residents.

**Trial registration:**

The trial was prospectively registered at DRKS.de with the registration number DRKS00014957 on October 9, 2018.

## Background

The worldwide population is progressively aging, which is why an increased demand for long-term care is expected [1]. Aging is associated with a decline in physical and cognitive functioning as well as with an increased occurrence of adverse health events. Consequently, the prevalence of disabilities increases substantially in the aging population, particularly after the age of 85 [2]. The condition of old people living in nursing homes is often characterized as multimorbidity at high risk of disability onset or progression [3]. This might lead to a loss of independence in activities of daily living (ADL), which is often closely associated with institutionalization and death [4]. Moreover, the declining physical functional status affects the overall quality of life of older adults [3]. Thus, effective interventions to strengthen health resources and prevent or delay disabilities and the loss of physical and cognitive functioning in older institutionalized people is a public health priority [3].

With the German Prevention Act of 2015, German nursing care insurances must provide preventive services in nursing homes that are aiming at promoting the health of residents by maintaining or improving several domains, such as physical functioning and mobility, cognition and quality of life [5]. The PROCARE project uses the BASE-program [6] to provide a strategy for the health promotion process described in the prevention guidelines [5].

The current project focusses on improving the above-mentioned goals. Therefore, a multicomponent exercise intervention program that takes the desires and preferences of the residents into account will be conducted. There is strong evidence that structured exercise programs in healthy and pre-frail older individuals can effectively improve everyday functionality, mobility, while reducing falls and physical frailty [7–12]. In addition, the positive effect of regular physical activity on cognition and on the prevention of diseases (such as cardiovascular diseases, diabetes, osteoporosis or sarcopenia) has already been demonstrated [1, 13–16]. In contrast, evidence from exercise interventions in the nursing home setting is less clear and inconsistent. A systematic review [17] showed that intervention studies in very frail and multimorbid populations cannot support the beneficial effect of exercises on functional performance and hence suggest that the degree of frailty might be critical, when reviewing the effectiveness of exercising [17]. Confirming these findings, a study with a moderate intensity group-exercise program positively influenced the reduction of falls and improved physical performance in pre-frail, but not in frail elderly nursing home residents [18]. Contrary to the findings in community-dwelling older individuals [18–20], exercise interventions have not been able to reduce falls in nursing homes [21]. Nevertheless, a few studies showed a positive impact of exercise on ADL [1, 16] and functional capacity [12, 16, 22–24] for people living in nursing homes. A systematic review investigating frail older people in nursing homes, residential care, and in the community [25] demonstrated that most studies provide evidence that exercise interventions have a positive impact on frailty. However, the definition of the term ‘frailty’ was different and unclear among most studies. Moreover, in the majority of studies, effect sizes were small [1] and a clear recommendation for an appropriate intervention was not given.

For cognitive outcomes and dementia, several studies with nursing home residents indicated no differences between exercise and cognitive intervention groups compared to the control group [26–31]. However, the findings also demonstrate a prominent heterogeneity regarding type, duration and frequency of exercise and severity of participants’ dementia. On the other hand, some studies addressing physical training and exercises reported positive effects on cognitive performance (short-term memory recall, visuospatial abilities, multiple aspects of executive functions) in the setting of nursing homes [1, 22, 23, 32–34]. The analysis of previous studies [1, 22, 23, 26–34] indicates that programs using higher intensities (e.g., walking exercises with additional weights) and longer training periods (> 3 months; at least twice a week [35]) tend to have a greater impact on cognitive performance. Moreover, most benefits on motor and cognitive performance seem to be reached by dual-task training interventions [35].

For quality of life, exercise interventions have shown to improve older adults’ well-being [23, 36], particularly depressive symptoms were reduced in people with dementia [37]. However, a large RCT, aimed at reducing depressive symptoms to increase well-being among nursing home residents, conducted a moderately intense exercise program twice a week for 12 months and found no effect [38]. A high-intensity functional exercise program aiming to reduce depressive symptoms and improve psychological well-being showed no effect among older people living in residential care facilities, but positive effects among people with dementia [36]. Nevertheless, there is neither consistent evidence, nor evidence-based physical activities, nor exercise guidelines to promote health-related outcomes (like physical and cognitive functioning and quality of life) for very old, multimorbid, and institutionalized people [39–41]. Despite the insufficient evidence, a recent report from the International Association of Gerontology and Geriatrics - Global Aging Research Network (IAGG-GARN) and the IAGG European Region Clinical Section provides first recommendations for physical activity in older persons, who are in need of care [41]. To lower the risk of developing a number of disabling medical conditions and various chronic diseases, they propose a multicomponent program administered in small groups, including training of strength, endurance, mobility, and balance, in combination with dual-task exercises in moderate intensities twice a week for 35–45 min each. The report also emphasizes continuously adapted training intensities in relation to the residents’ abilities. Also, progressive enhancement, inclusion of stimulating materials and music, as well as training for movements that are often associated with falls (e.g., walking forward with changes of direction) is advised. In addition, the preferences and needs of the individuals should be discussed in advance, in order to define feasible goals and to take the residents’ self-efficacy into account [42].

Overall, the effectiveness of preventive interventions in nursing homes which address these recommendations and cognitive-motor exercises is assumed, but not yet examined. Thus, more high-quality studies are needed to examine and to structure results of preventive interventions, so they can be implemented into the health care system [42].

### Aims and research questions for the study

Based on the existing research and the physical activity recommendations mentioned above, a multicenter intervention study will be conducted, aiming to determine the feasibility and efficacy of a multicomponent exercise intervention program for residents of nursing homes. Moreover, we assume that these effects will improve the residents’ quality of life.

## Methods/design

The SPIRIT statement [43] was used as a guideline for this protocol paper.

### Trial design

This study is a two-arm single-blinded randomized controlled trial of an individually tailored multicomponent intervention (see Table 1) for older men and women living in nursing homes. The study will be aptly named PROCARE – Prevention and occupational health in long-term care, as part of the PROCARE project. A stratified randomization is performed after the baseline assessment. The assessment of primary and secondary outcomes takes place in all subjects upon entry to the study (T1) by a blinded assessor and is repeated at 16 weeks (T2), and at 32 weeks (T3, retention test, intervention group only) after randomization (see Table 2). *The trial is registered at DRKS.de with registration number* DRKS00014957.

**Table 1:** Description of the intervention

Program	Week 1–4	Week 5–8	Week 9–12	Week 13–16
Mobilisation and warm-up	e.g., range of motion exercises for the wrists, hip, shoulders, knees, and ankles	Cf. week 1–4	Cf. week 1–4	Cf. week 1–4
Coordination, balance, and cognitive exercises	e.g., standing balance, bodyweight shifting, motivational cognitive-motor games with group interaction including balls and scarfs	e.g., standing balance, bodyweight shifting, motivational cognitive-motor games with group interaction including balls and scarfs	e.g., standing balance with feet together, side-by-side, bodyweight shifting, motivational cognitive-motor games with group interaction including balls and scarfs	e.g., standing balance with feet together, side-by-side, semi-tandem, tandem, standing on one leg, bodyweight shifting, motivational cognitive-motor games with group interaction including balls and scarfs
Dual-task walking exercises (endurance)	150-180 m e.g., brisk walking, starting, stopping, avoiding obstacles, turns	180-240 m e.g., brisk walking, starting, stopping, avoiding obstacles, turns	240-300 m e.g., brisk walking, starting, stopping, avoiding obstacles, turns, dual-task conditions e.g., carrying a cup, repeating rows of numbers, paying attention to signs	300-330 m e.g., brisk walking, starting, stopping, avoiding obstacles, turns, dual-task conditions e.g., carrying a cup, repeating rows of numbers, paying attention to signs
Calm down	e.g., stretching and relaxing exercises	Cf. week 1–4	Cf. week 1–4	Cf. week 1–4

**Table 2:**
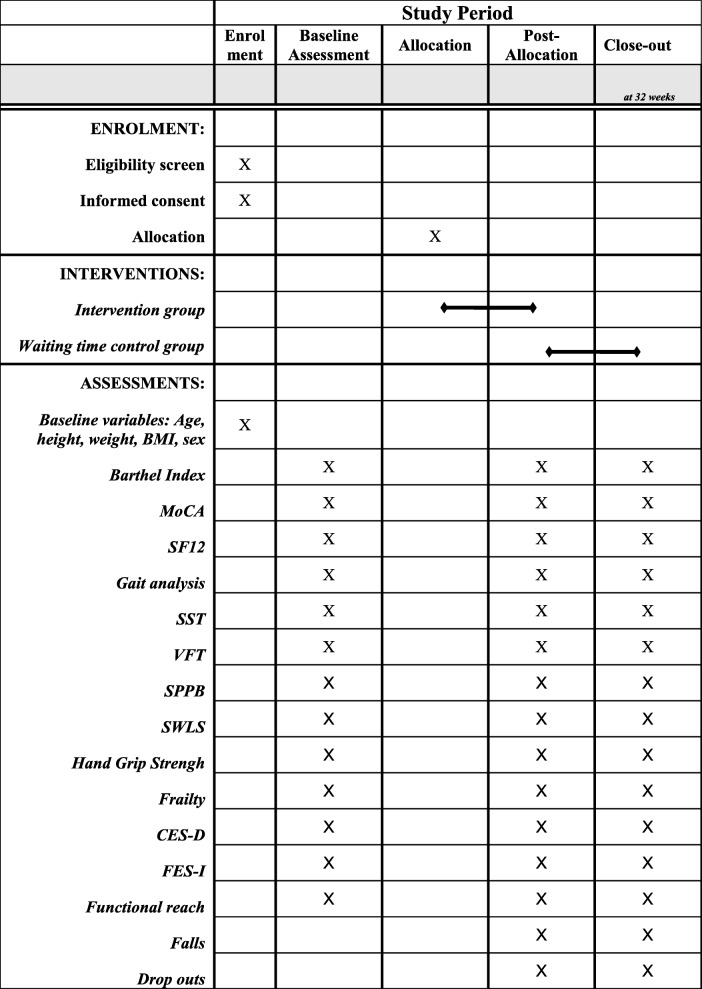
Schedule of enrolment, interventions, and assessments

### Participants, interventions, and outcomes

#### Ethics approval

The trial is conducted in agreement with the principles of the Declaration of Helsinki and the guidelines of Good Clinical Practice (GCP). All participants or their legal guardians give written informed consent prior to the study enrollment. The ethics committee of the Hamburg Chamber of Physicians, Germany, has approved the study protocol *(PV5762).*

#### Recruitment of nursing homes

Eight cities and their surroundings throughout various regions in Germany (Bremen, Chemnitz, Frankfurt, Hamburg, Karlsruhe, Nuremberg, Paderborn and Stuttgart) will recruit 48 nursing homes in total (six per site). The institutions involved are deliberately selected based on their basic structural figures (number of nursing places, number of employees, urban or rural district, social-economic status), in order to analyze the applicability of the program under a wide range of conditions. Therefore, a list of all nursing homes will be created. It will then be stratified by their structural characteristics and afterwards the nursing homes will be randomly selected. Participation is voluntary and will not be remunerated. In case a requested nursing home declines to participate, another facility with similar characteristics will be requested instead.

#### Recruitment of participants

Assessment of eligibility and recruitment of participants with respect to inclusion and exclusion criteria will be primarily based on nursing documentation and staff consultation. Care management and lead investigators will meet to discuss and create a list with suitable nursing home residents, prior to the study enrollment. It will be made clear that the intervention is targeting everyone who meets the inclusion criteria and not only those who are very open to physical activities and therefore might be more likely to show a positive response to the intervention. Even the very institutionalized, frail residents will be encouraged to participate. Nevertheless, it is a voluntary intervention and there is still room for bias because the individual reasons for participation will remain unclear. After the selection process, nursing staff will inform all suitable residents or their legal guardians about the study goals and ask for voluntary participation. All verbally consented participants or their legal guardians will give written informed consent prior to the study enrollment.

#### Eligibility criteria

Inclusion criteria are *i)* willingness to participate, *ii)* ability to participate in group activities, *iii)* ability to sit unassisted on a chair or in a wheelchair, and *iv)* the ability to understand and execute simple instructions. No other inclusion or exclusion criteria will be applied.

#### Assignment of interventions

Assessment and data collection will be done by blinded assessors in a strictly pseudonymized form to guarantee a blinded data analysis.

To avoid performance bias, the measurements and the intervention follow a standardized protocol. All participant information and data will be stored securely and identified by a coded ID number only to maintain the participants’ confidentiality.

To avoid selection bias, a stratified randomization will be conducted to divide the participants into either an intervention group or a waiting time control group. The random allocation will be stratified and executed by lot by the director of the study who will receive the pseudonymized codes of the participants and their baseline characteristics and will not be involved in neither the assessment nor the intervention procedures. Stratification will be based on comparable sex, age, and cognitive performance (according to the Montreal Cognitive Assessment MoCA-Score [44]) to avoid differences in the baseline characteristics of the groups. After assigning the participants to either the intervention or waiting time control group, the pseudonymized participant codes will be sent to the study investigators who are responsible for the data management. The exercise scientist or physiotherapist, who will conduct the intervention, will receive only names of the participants in the intervention group and after 16 weeks names of the waiting time control group without being aware of the control group design.

### Outcome measures

The assessment will focus on three key domains: physical functioning, cognitive performance, and psychosocial well-being. Apart from the following primary and secondary outcomes, demographic and baseline characteristics, like age, height, weight, Body Mass Index, and sex, will be measured.

#### Primary outcomes

The following primary outcomes will be measured to evaluate the efficacy of the intervention program:

##### Physical functioning

The *Short Physical Performance Battery* (SPPB) [45] is a standardized instrument to test the lower extremity functionality (balance, gait speed, leg strength). Participants are required to show a stable stand in an upright position under three conditions (legs closed/feet together, semi-tandem stand, tandem stand). After that, comfortable gait speed will be assessed by measuring the time to walk a 4-m track, starting from a standing position and stopping when the first foot is past the 4-m line. Finally, a five times sit-to-stand transfer will be completed as fast as possible. Each domain is scored between 0 and 4 and SPPB overall scores range from 0 (low mobility/functionality) to 12 (full mobility/functionality). Clinically relevant improvements have been demonstrated to range between 0.99 and 1.34 points for the SPPB [45].

*Gait analysis* (GAITRite: CIR Systems Inc., Clifton, NJ, USA, Optogait: Microgate, Bolzano, Italy, MobilityLab: APDM Inc., Portland, USA, GaitUp: SA, Lausanne, Switzerland or Zebris PDM, Isny, Germany). Gait performance will be assessed by measuring step length, step width, gait speed, and double support phase on a 10-m track, using of one of the mentioned gait analysis systems. Each participant completes three trials: a test trial, one trial at preferred walking speed, and one trial at maximum walking speed. Measured data will be recorded and saved for later analysis by the gait systems software. An accompanying validation study will secure the comparability of the different gait measurement systems.

##### Dual-task cognitive performance

The *Serial Sevens Test* (SST) [46] aims to assess cognitive functioning. During the SST, participants are being asked to count down from a certain number in steps of seven. Due to the poor cognitive functioning of most participants, a simpler version of the SST will be administered, in which participants have to count down in steps of 3 (S3T) and steps of 1 (S1T). The S3T and S1T will be tested during a single- and dual-task condition (i.e. during gait), in order to evaluate the cost of dual-tasking for cognitive functioning. The number of correct answers within 15 s will be recorded during single- and dual-task conditions as well as the gait parameters step length, step width, gait speed, and double support phase under dual-task conditions with a gait analysis system.

The *Verbal Fluency Test* (VFT) is an additional test for cognitive functioning and part of the MoCA [44]. The VFT is a phonemic fluency test, in which participants are asked to name as many words as possible in a certain time, starting with a specific letter (not allowed are names or numbers). It was shown that verbal fluency is reduced in elderly people with mild cognitive impairments as compared to their non-impaired peers [47]. In accordance to the S3T and S1T, the VFT will be administered during single- and dual-task conditions, to evaluate the cost of dual-tasking for cognitive functioning. The number of correct answers within 15 s will be recorded during single- and dual-task conditions as well as the gait parameters step length, step width, gait speed, and double support phase under dual-task conditions with a gait analysis system.

##### Psychosocial well-being

The short form of the *Health Survey SF12* [48] is a questionnaire, which can be used to examine the health-related quality of life of the participants, who rate their quality of life via twelve items. The items regard eight health concepts which are commonly represented in widely used surveys: (1) physical functioning, (2) role limitations due to physical health problems, (3) bodily pain, (4) general health, (5) vitality (energy/fatigue), (6) social functioning, (7) role limitations due to emotional problems, and (8) mental health (psychological distress and psychological well-being) [49]. The SF12 physical and mental component summary scales are scored using norm-based methods. Both scales are transformed to have a mean of 50 and a standard deviation of 10 in the general U.S. population. All scores above and below 50 are above and below the average [49].

The *Satisfaction with Life Scale* SWLS [49] is a brief instrument with five items to measure global cognitive judgements of satisfaction with one’s life on a seven-point Likert scale. High scores indicate a high satisfaction with life, while low scores indicate a low degree of satisfaction.

#### Secondary outcomes

The following secondary outcomes will be measured to evaluate the efficacy of the intervention program:

##### Physical functioning

The *Barthel Index* [50] is used to systematically record the independence of participants when performing basic ADL via ten items. Feeding, personal toileting, bathing, dressing and undressing, getting on and off a toilet, controlling bladder, controlling bowel, moving from wheelchair to bed and returning, walking on level surface (or propelling a wheelchair if unable to walk) and ascending and descending stairs are rated on a scale from 0 to 15 points depending on the item. Total possible scores range from 0 (totally dependent) to 100 (fully independent) [50].

*Hand Grip Strength* is measured with a hydraulic hand dynamometer (JAMAR, hydraulic hand dynamometer). Three trials with each hand will be executed. Results will also be used to assess the frailty index item weakness.

The *Functional reach* [51] is a clinical measure of balance. It assesses the difference between the arm’s length and maximal forward reach in cm, using a fixed base of support. The test will be executed in a sitting position. The participant sits against the back of a chair next to a wall reaching forward as far as possible without losing balance. Reach distance will be measured with a scale attached to the wall.

For the measurement of *Frailty* this study will apply the original operationalization of the Frailty Phenotype from the Cardiovascular Health Study [52] to enable comparability. A frailty index will be formed out of five measured factors, including an unintentional weight loss of more than 4.5 kg in the past 12 months (shrinking), BMI- and sex-adjusted hand grip strength (weakness), frequency of fatigue in the last week by using two items of the Center of Epidemiological Studies-Depression Scale (CES-D, exhaustion), height- and sex-adjusted gait speed (slowness) and sex-adjusted energy expenditure by physical activity (modified Minnesota Leisure Time Physical Activity Questionnaire).

The short form of the *Falls Efficacy Scale-International* (Short-FES-I) [53] is a seven-item questionnaire with a scoring range between one and four, which provides information on the level of concern about falls for a range of activities of daily living. The number of falls, fall-related injuries and deaths occurring during the 32-week period will be documented by nursing staff.

##### Cognitive performance

The *Montreal Cognitive Assessment* (MoCA) [44] is a brief screening tool of global cognition to reveal mild cognitive impairment and an early stage of Alzheimer’s disease. It assesses several cognitive domains, like short-term memory recall, visuospatial abilities, multiple aspects of executive functions, attention, concentration and working memory, language and orientation to time and place. MoCA scores range between 0 and 30. A score of 26 or above is considered to be normal [44].

##### Psychosocial well-being

The *Center for Epidemiological Studies Depression Scale* (CES-D) [54] is used to screen for depressive symptoms and mood disorders with an eleven-items questionnaire, scoring between zero and three points for each item. It has demonstrated validity for research conducted in elderly populations [55]. Two items regarding the exhaustion of residents (“Everything was effort”; “I could not get going”) will also be used to assess the ‘exhaustion’ for the frailty index.

### Intervention

The exercise program consists of 32 sessions for a period of 16 weeks. One training session has a duration of 45–60 min and takes place twice a week. Exercise sessions will be administered by at least one certified exercise scientist or physiotherapist with group sizes ranging from four to 15 participants. The program follows IAGG guidelines and combines previously published exercises that have proven to be beneficial for cognitive-motor performance in older people in the community and in need of care [7, 8, 12, 20, 35, 56–59]. Training focusses on daily situations which are commonly associated with an increased fall risk and it mostly includes challenging walking exercises (e.g., brisk walking, starting, stopping, avoiding obstacles, turns). During these exercises, participants are also exposed to a variety of cognitive tasks under single- and dual-task conditions, designed to challenge their focus of attention with acoustic and visual stimuli and specific executive functions. Furthermore, exercises for strength, balance and flexibility as well as endurance performance associated with walking are integrated.

To ensure a controllable structure, training sessions are divided in five parts: 1. 5–10 min mobilisation and warm-up (e.g., range of motion exercises for the wrists, hip, shoulders, knees, and ankles). 2. 10 min coordination, balance, and cognitive exercises (e.g., standing balance, bodyweight shifting, motivational cognitive-motor games with group interaction including balls and scarfs). 3. 20 min aerobic walking exercises (e.g., under different single and dual-task conditions). 4. 10 min strength exercises (e.g., chair rises, upper body and trunk exercises with additional materials and weights, functional lower-limb exercises). 5. 5–10 min calm down (e.g., stretching and relaxing exercises).

During the first step of the conceptualization, qualitative guided interviews were conducted with five residents of a nursing home facility. The interviews assessed different domains regarding ADL, need of support, participation in social activities as well as expectations and wishes regarding a training program. Moreover, a feasibility study (currently under review) was conducted to examine the adherence and acceptance of the program. Taking into account these previously inquired desires and preferences of the residents, a focus is set on everyday skills to promote ADL, cognition and psychosocial resources. For example, by using motivational equipment with different colors and music during the exercise sessions, a stimulating environment will be provided to promote participant’s retention. The exercise program will be continuously adapted to the residents’ capacity and hence, it is organized as a progressive challenge to expand participants’ resources in accordance to the F.I.I.T. principle [60]. The intensity of exercises will vary between moderate and vigorous. This will be ensured by adjusting the duration, frequency, difficulty, range of motion and/or intensity of the exercises. For example, endurance exercises, like 15 m walks, will range from ten up to 22 walks within one session. For residents who are unable to walk a program will be conducted with exercises exclusively in a sitting position. Regarding resistance exercises, progression will be ensured by adjusting the number of repetitions (from 5 to 10 to 15 to 20), the number of sets (1, 2 or 3), and/or by usage of additional weights (1 or 2 kg). For executing static, dynamic balance, and coordination exercises the difficulty level will be raised by changing exercise positions (e.g., sitting, standing, feet together, side-by-side, semi-tandem, tandem, standing on one leg). To assess the intensity of training, instructors will use the Borg Scale of Perceived Exertion [61].

Discontinuation of the intervention may occur in case of health decline or if a participant wishes to stop taking part in the group intervention. To improve adherence and to promote retention, the therapists will give explanations about the purpose of the intervention and the possible benefits of the exercises. Attendance of each participant will be recorded and reasons for drop outs will be documented. No other concomitant group exercise interventions are permitted besides usual care and physiotherapy. Control group participants will be asked to continue their regular everyday activities.

### Data collection, management, and analysis

#### Statistical analysis

We will evaluate the effects of the intervention on every quantitative, qualitative, and ordinal outcome, using repeated measures or mixed models, “t” tests, Kruskall-Wallis Mann-Witney tests or the Chi-square tests, depending on the type of outcome and their normal or non-normal distribution. The primary analysis will be a mixed model between-group comparison of the SPPB, gait variables, Serial Sevens, and Verbal Fluency Test, utilising all available data points during follow-up. We will use the Bonferroni correction to appropriately adjust the overall level of significance for multiple comparisons. Between-group differences for all primary and secondary outcomes will be adjusted for baseline values, age, sex, and education. Secondary outcomes will be analysed with similar methodology, using repeated measures mixed model between-group comparisons. All statistical analyses will be performed using SPSS Statistics for Windows (version 25.0, IBM). Statistical significance level is set at *p* < 0.05.

Intention-to-treat analysis will be performed (participants who are randomized into groups after the collection of baseline data). For the intention-to-treat analysis, data of all trial patients in the groups to which they were randomized will be processed, regardless of whether they received or adhered to the allocated intervention. It is assumed that the majority of participants in the two arms will receive the appropriate number of intervention sessions. In addition, a per-protocol analysis of the participants who completed the study without major protocol violation (e.g., who attended more than 80% of the training sessions), will be performed. The per-protocol analysis will be performed as a secondary analysis, if there is a sufficient number of participants in the two arms, who do not receive the intervention protocol or are lost to outcome assessment. Data from those participants, who do not violate the treatment protocols, will be included in the per-protocol analysis. The multiple imputation (MI) technique will be used for dealing with missing data under the assumption that data are missing at random.

#### Sample size estimate / power calculations

The required sample size was calculated with G*Power (Version 3.1.9.2, Heinrich Heine University of Duesseldorf) [62]. The sample size calculation was approximated with a 2 × 3-factorial analysis of variance (ANOVA) for repeated measures (within-between interaction, small effect size, power of .80 [1-β], 2-sided α-error (95% CI), 2 groups, 3 measurements) based on the primary outcome Short Physical Performance Battery (SPPB). The small effect size used for the calculation of required sample size is based on literature reviews and assumptions of clinically relevant changes for residents in nursing homes with probable cognitive impairment [63]. One hundred eight individuals per region are required in order to detect a clinically meaningful change of ≥1.0 point with a SD of 0.99 points. To account for potential dropouts before study completion, we will inflate the sample size by 30% (20% losses during follow-up; 10% mortality), resulting in a total sample size of 1120 individuals (140 per centre with 70 participants allocated to each group).

#### Monitoring

A data monitoring committee, responsible for data monitoring, interim analyses and auditing, will not be established, because no adverse events are to be expected. However, study participants will be under the surveillance of trained project staff, who will intervene, if a negative reaction is observed during the measurements and training interventions. Nevertheless, grant holders are part of a PROCARE advisory board and responsible for data audits every 5 months.

#### Dissemination

The results of the study will be published in open-access and international journals. In addition, the results will be presented at conferences as well as in the participating nursing homes.

## Discussion

To determine the efficacy and feasibility of a multicomponent exercise intervention for nursing home residents, a multicenter intervention study will be conducted. We assume improvements or a slower decline of frail and pre-frail residents’ physical and cognitive functioning as well as psychosocial well-being compared to a waiting time control group.

Preventive physical activity interventions could preserve the health-related quality of life of nursing home residents, since a reduction is based particularly on a loss of physical functioning [41]. We propose, that nursing home residents with severe physical and cognitive impairment might benefit from participation in physical activity interventions, because of their low functional status at the beginning and a higher physiological adaptation to a progressive training intensity [41].

There are only vague guidelines for the content, intensity, frequency, and duration of physical activity in the nursing home setting [40], yet. High-quality studies are required to close this gap and provide effective and efficient exercise modalities for this setting. The results of the present study will yield recommendations for exercise interventions, which then can be implemented into the health care system.

The intervention of this study program combines components of exercise programs that have proven to gain health benefits for residents in nursing homes [1, 12, 16] in residential care [7, 56], and in older adults living in the community [20, 35, 57–59], with a special focus on cognitive-motor exercises. Furthermore, based on this multicomponent program with strength, balance, and dual-task components, the findings will help to derive valid recommendations for activities and guidelines for health promotion in nursing home residents. Results from this trial will particularly contribute to the evidence on cognitive-motor approaches in the maintenance of mental and physical functioning. It will also offer potential ways to encourage nursing home residents to participate actively in social life within the care setting, by providing a program that is appropriate and adapted to residents’ capacities, needs and desires. To this end, the findings may provide suggestions and support to deal with present and future challenges, occurring at health promotion initiatives in the setting of nursing homes, a sector that likely will gain more relevance in times of the demographic change. With the Prevention Act of 2015, German health insurances have to provide preventive services in nursing homes [5]. The trial will show that universal prevention through physical activity interventions in this setting in late life care is possible and useful to improve health status and personal resources of nursing home residents.

## Data Availability

Data can be obtained from the corresponding author upon reasonable request.

## Abbreviations

ADL: Activities of daily living
BMI: Body Mass Index
CES-D: Center for Epidemiological Studies Depression Scale
FES-I: Falls Efficacy Scale international
GCP: Good Clinical Practice
GDS: Geriatric Depression Scale
MoCa: Montreal Cognitive Assessment
PROCARE: Prevention and occupational health in long term care
S1T: Serial 1 Test
S3T: Serial 3 Test
SD: Standard deviation
SF 12: Short Form (12 questionnaire) SF 36 Health Survey
SPPB: Short Physical Performance Battery
SST: Serial Sevens Test
SWLS: Satisfaction with Life Scale
TWT: Trail Walking Test
VFT: Verbal Fluency Test
